# Evaluating the exploratory classification performance of multimodal magnetic resonance imaging in neuropsychiatric systemic lupus erythematosus: a prospective cohort study

**DOI:** 10.3389/fnins.2026.1773928

**Published:** 2026-03-17

**Authors:** Haoyu Wang, MengFan Sun, Xiaojuan Yang, Sha Li, Shiling Zhou, Xiaoyao Lin, Qiyuan Zhu, Jun Liu

**Affiliations:** 1 Department of Medical Imaging, Chongqing Emergency Medical Center, Chongqing University Central Hospital, School of Medicine, Chongqing University, Chongqing, China; 2 Department of Rheumatology and Immunology, Chongqing Emergency Medical Center, Chongqing, China; 3 Department of Medical Imaging, The First Affiliated Hospital of Chongqing Medical University, Chongqing, China; 4 Department of Medical Imaging, Chongqing Emergency Medical Center, Chongqing, China

**Keywords:** diffusion tensor imaging analysis along the perivascular spaces, high-resolution vessel wall imaging, magnetic resonance imaging, Normalized Wall Index, systemic lupus erythematosus

## Abstract

**Objectives:**

To investigate the associations between magnetic resonance imaging (MRI) quantitative parameters with neuropsychiatric systemic lupus erythematosus (NPSLE), and to evaluate the exploratory classification performance for NPSLE.

**Methods:**

This study recruited 60 systemic lupus erythematosus (SLE) patients and 20 healthy controls (HC). Participants underwent multimodal MRI and clinical assessment. The diffusion tensor imaging analysis along the perivascular spaces value (DTI-ALPS) and Normalized Wall Index (NWI) were derived using Diffusion Tensor Imaging (DTI) and high-resolution vessel wall imaging (HR-VWI). MRI quantitative parameters were analyzed using Kruskal-Wallis test and Dunn’s test pairwise comparisons. Partial correlation analysis was conducted on MRI parameters and clinical data. An exploratory multimodal classification model was developed.

**Results:**

The NPSLE group showed lower DTI-ALPS values and lumen area (LA), and higher NWI compared to the other groups (*P* < 0.01). After adjusting for age, gender, and body mass index (BMI), DTI-ALPS correlated negatively with total cholesterol (TC) and immunoglobulin A (IgA) (*r* = –0.308, *P* = 0.025; *r* = –0.301, *P* = 0.028). LA correlated negatively with low-density lipoprotein (LDL) and disease duration (*r* = –0.293, *P* = 0.032; *r* = –0.474, *P* < 0.001). The exploratory multimodal classification model combining clinical data, white matter hyperintensities (WMH) status, and MRI parameters showed excellent classification performance (area under the curve (AUC) = 0.933).

**Conclusion:**

NPSLE patients exhibited low DTI-ALPS values and high NWI compared to other groups. The exploratory model based on multimodal MRI quantitative parameters may provide imaging insights into cerebrovascular alterations and glymphatic dysfunction associated with NPSLE.

## 1 Introduction

Systemic lupus erythematosus (SLE) is an autoimmune disease that affects multiple systems, with an incidence rate of approximately 1.4–11 cases per 100,000 people, predominantly affecting females (Aibara et al., 2018; Stojanovich et al., 2007). The pathophysiological mechanisms include autoimmunity, vascular lesions, neuroinflammation, blood brain barrier (BBB) disruption, and degenerative changes (Zucchi et al., 2023). SLE presents with a wide range of clinical symptoms, potentially affecting the central nervous system, urinary system, digestive system, hematological system, respiratory system, cardiovascular system, and peripheral joints. Neuropsychiatric systemic lupus erythematosus (NPSLE) is one of the most severe complications of SLE, with clinical manifestations encompassing 19 neuropsychiatric symptoms defined by the American College of Rheumatology (ACR AD HOC Committee on Neuropsychiatric Lupus Nomenclature, 1999). These range from headaches and mild cognitive impairments (such as attention deficits and memory decline) to severe neurological disorders (such as seizures, cerebrovascular events, and psychiatric symptoms) (Zucchi et al., 2023), characterized by nonspecific clinical manifestations and high mortality, making it one of the most challenging complications of SLE (O’Neill and Cervera, 2010). Due to the non-specific nature of NPSLE’s clinical presentations and the lack of objective biomarkers to assess nervous system in NPSLE patients (Clark et al., 2017), early diagnosis often lags behind pathological progression, leading to delayed treatment.

Magnetic resonance imaging (MRI) is widely used for neuroimaging assessment in NPSLE (Luyendijk et al., 2011). Currently, MRI research predominantly focuses on white matter changes in NPSLE patients. However, vascular inflammation in NPSLE patients can lead to thickening of vessel walls and lumen narrowing (Ide et al., 2018). These changes may be closely associated with disease activity in SLE and the onset of neuropsychiatric symptoms (Cohen et al., 2017). The brain glymphatic system plays a crucial role in transporting nutrients and maintaining the cerebral homeostasis. Neuroinflammation may impair glymphatic function, reducing the exchange of substances and waste clearance across the BBB (Oshio, 2023). The diffusion tensor imaging analysis along the perivascular spaces (DTI-ALPS) technique enables evaluation of glymphatic system function (Iliff et al., 2012). Dyslipidemia is common in SLE and is associated with inflammation-related vascular injury and increased atherosclerotic risk (Szabó et al., 2017). Given the involvement of neurovascular mechanisms and small vessel disease in NPSLE, lipid metabolism biomarkers were included as readily available clinical indicators to explore their associations with MRI quantitative parameters, including the DTI-ALPS index, which reflects glymphatic and perivascular function (Faria et al., 2017). Therefore, multimodal MRI may offer valuable insights into the underlying neurobiological mechanisms of NPSLE.

This study combines Diffusion Tensor Imaging (DTI) and high-resolution vessel wall imaging (HR-VWI) to investigate alterations in cerebral vascular microstructure and glymphatic system function in patients with NPSLE. In HR-VWI image analysis, the M1 segment of the middle cerebral artery was selected for evaluation because it supplies a core cerebral perfusion territory and has been reported to be associated with cognitive impairment and neuropsychiatric manifestations in NPSLE (Zhao et al., 2025). In addition, owing to its favorable imaging visibility, the M1 segment allows clear delineation of the vessel wall and lumen on HR-VWI, making it particularly suitable for quantitative assessment (Ide et al., 2018). This study aimed to examine between-group differences in quantitative neuroimaging parameters and to investigate the associations between clinical characteristics, laboratory findings, and quantitative imaging metrics using partial correlation analyses. Furthermore, by integrating clinical and imaging variables, we tested multiple classification exploratory models to evaluate the classification performance of quantitative MRI parameters in NPSLE.

## 2 Materials and methods

### 2.1 Patients and clinical features

This study is a prospective investigation approved by the Ethics Committee of the Chongqing University Affiliated Central Hospital (Municipal Emergency Center Hospital), with the approval number 2025 Ethics Review No. 98. Written informed consent was acquired from all participants before the study commenced. We recruited patients with SLE from the Rheumatology Department of Chongqing University Affiliated Central Hospital outpatient clinic and through community recruitment from July 2024 to July 2025. Inclusion criteria for the study included patients diagnosed with SLE based on the 1997 American College of Rheumatology classification criteria or the 2012 Systemic Lupus International Collaborating Clinics classification criteria (ACR AD HOC Committee on Neuropsychiatric Lupus Nomenclature, 1999), within the age range of 18–60 years. Exclusion criteria included a history of cerebral infarction, encephalitis, or intracranial tumors; pre-existing primary psychiatric disorders diagnosed prior to the onset of SLE; and conditions incompatible with MRI examination, such as claustrophobia or metal implants. Based on the 19 neuropsychiatric symptoms proposed by Systemic Lupus International Collaborating Clinics and a multidisciplinary consensus, patients were categorized into Non-NPSLE and NPSLE groups, with collaboration from both the Rheumatology and Psychiatry departments. Importantly, group allocation was established through multidisciplinary clinical consensus during the current disease course prior to image analysis, ensuring that imaging findings did not influence NPSLE classification. Clinical data collected in this study included age, sex, body mass index (BMI), handedness, disease duration of SLE, initial SLE manifestations, presence of neuropsychiatric symptoms at disease onset, medication use Information on medication use was collected for all patients, including corticosteroids and immunosuppressive agents. Corticosteroid use was categorized as high-dose or low-dose based on a daily prednisone-equivalent dose greater than 10 mg. Immunosuppressive agents were classified as either used or not used. Medication use was included as a covariate in the statistical analyses to control for potential confounding effects. Laboratory parameters comprised antinuclear antibodies (ANA), anti-Smith (anti-Sm) antibodies, anti–double-stranded DNA (anti-dsDNA) antibodies, anti-nucleosome antibodies (ANua), anti–ribonucleoprotein (anti-RNP) antibodies, high-density lipoprotein (HDL), low-density lipoprotein (LDL), total cholesterol (TC), triglycerides (TG), complement components C3 and C4, and immunoglobulin levels including immunoglobulin A (IgA), immunoglobulin G (IgG), immunoglobulin M (IgM), and immunoglobulin E (IgE). Quantitative neuroimaging parameters included DTI-ALPS index, wall area (WA), wall thickness (WT), lumen area (LA) and NWI. Disease activity was assessed using the Systemic Lupus Erythematosus Disease Activity Index (SLEDAI). Additionally, 20 healthy control (HC), matched for age and sex, were recruited for comparison.

### 2.2 MR imaging acquisition

MRI was performed using a 3T Siemens scanner (MAGNETOM Skyra, Siemens, Erlangen, Germany) with a 64-channel head-specific coil. The scanning sequences included T1-weighted imaging (T1WI), T2-weighted imaging (T2WI), T2-weighted fluid-attenuated inversion recovery imaging (T2-FLAIR), three-dimensional T1-weighted imaging (3D-T1), three-dimensional-time-of-flight magnetic resonance angiography (3D-TOF MRA), DTI and HR-VWI. DTI was performed with the following parameters: voxel size 2.0 × 2.0 × 2.0 mm, slice thickness 2.0 mm, field of view (FOV) = 256 mm, 66 slices, repetition time (TR) = 7,100 ms, echo time (TE) = 72.0 ms. b-values = 1,000, b0 volumes = 10, number of directions = 64. The scan duration was 9 min and 8 s. Additionally, HR-VWI was using a T1-weighted vessel wall imaging sequence with intrinsic black-blood contrast for suppression of intraluminal blood signal. voxel size 0.7 × 0.7 × 0.7 mm, slice thickness 0.68 mm, FOV = 260 mm, 240 slices, TR = 700 ms, TE = 20 ms with a scan duration of 7 min and 23 s. 3D-TOF MRA was performed with the following parameters: TR = 21 ms, TE = 3.42 ms, flip angle = 18°, FOV = 250 mm, voxel size = 0.3 × 0.3 × 0.6 mm, slice thickness = 0.60 mm, 52 slices, and a total scan time of 6 min 31 s.

### 2.3 Image analysis and processing

**FIGURE 1:**
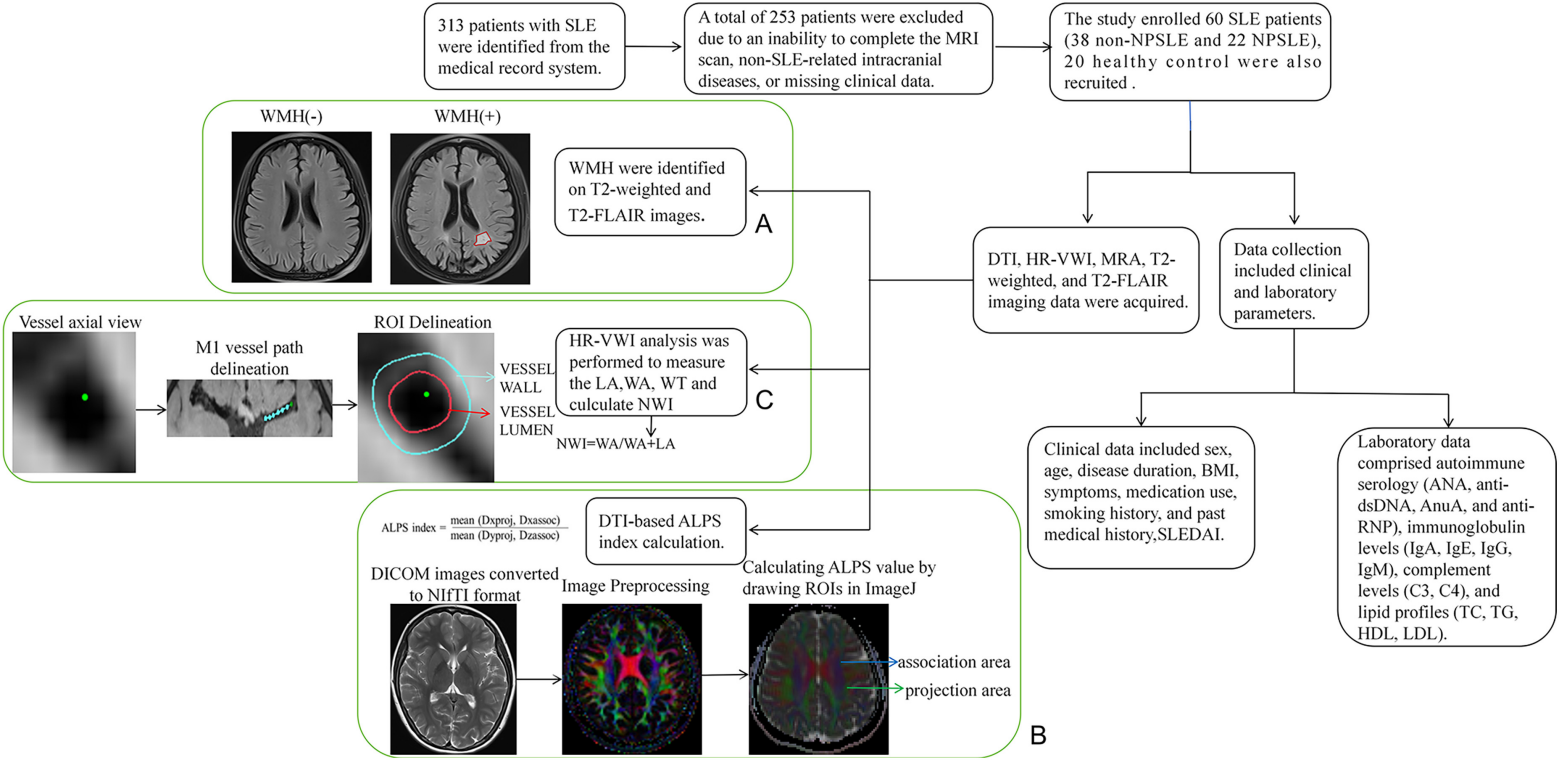
Flowchart of participant enrollment, group classification, MRI acquisition, and data analysis. **(A)** Identification of WMH (+) on T2-weighted and T2-FLAIR images. **(B)** Calculation of the DTI-ALPS index. **(C)** Measurement of vessel wall parameters on HR-VWI images.

The presence of white matter hyperintensities WMH (+) was assessed on T2 and T2-FLAIR images, and the results were classified into two categories (Figure 1A).

DTI image analysis and data processing were performed using internal codes from FMRIB Software Library. The preprocessing of images included format conversion of raw images using MRIcroGL, correction for eddy current distortions, motion artifact correction, denoising, gradient direction correction, brain tissue extraction, tensor fitting, and image registration (Clark et al., 2017; O’Neill and Cervera, 2010). The regions of interest (ROIs) were manually delineated on the Fractional Anisotropy (FA) map using ImageJ (1.53e, National Institutes of Health, United States), specifically locating the projection fibers and the association fiber regions at the level of the lateral ventricles, and measuring their diffusion coefficients in the x, y, and z directions (Figure 1B). ROIs were placed according to established DTI-ALPS protocols in projection and association fiber areas adjacent to the lateral ventricles (Mo et al., 2025), directionally oriented placement was used along the projection and association fiber directions at the level of the lateral ventricles to align with fiber orientation and better capture directional diffusivity characteristics, with minor adjustments made according to individual anatomical variations. The DTI-ALPS values were calculated (Yu et al., 2024). Higher DTI-ALPS values indicate better glymphatic system function (Oshio, 2023).

The HR-VWI images were analyzed and processed using internal software. The raw images were reconstructed in three orthogonal planes. The HR-VWI and 3D-TOF MRA images were aligned using anatomical landmarks to allow consistent vessel segment identification. The M1 segment of the middle cerebral artery was delineated based on 3D-TOF MRA and HR-VWI sequence images, ensuring that the path points were centrally located within the vessels. Surface reconstruction was performed based on the delineated vascular path, where the vessel wall and lumen could be differentiated according to signal intensity differences (Ide et al., 2018). The M1 segment on both sides of the same patient was delineated at three levels (with equal slice spacing between levels to ensure consistency). On the cross-sectional images of the surface reconstruction, the vessel lumen and wall of the bilateral M1 segment of the middle cerebral artery were outlined and further adjusted for details. Finally, the quantitative parameters WT, WA, LA, and NWI (NWI = WA / (WA + LA)) were calculated for analysis (Cai et al., 2005; Figure 1C). The research process is shown in Figure 1.

Two trained radiologists, Wang and Sun, independently performed image analysis and measured the imaging quantitative parameters. Both radiologists were blinded to the patient’s diagnosis, groupings, and each other’s measurement results to control for observer bias. The final quantitative measurements were obtained by averaging the values from the two radiologists. Intraclass Correlation Coefficient (ICC) was used to assess consistency.

### 2.4 Statistical analysis

Statistical analysis was performed using SPSS 26.0 and RStudio 4.5.1. A two-sided *P* < 0.05 was considered statistically significant. The normality of clinical information, laboratory data, and MRI quantitative parameters was assessed using the Shapiro-Wilk test. Categorical data were expressed as percentages, and normally distributed continuous data were presented as mean ± standard deviation (x ± s). Non-normally distributed continuous data were presented as median and interquartile range (IQR). For clinical data (age and gender) among the three groups, the Kruskal-Wallis test was used for inter-group difference analysis. Laboratory indicators between the Non-NPSLE and NPSLE groups were analyzed using the Mann-Whitney U test or chi-square test. MRI quantitative parameters among the three groups were analyzed using the Kruskal-Wallis test to assess inter-group differences, followed by pairwise comparisons using Dunn’s test, with Bonferroni correction applied. To eliminate the effect of scale, all continuous variables were standardized using Z-scores. Spearman correlation analysis was then performed to assess the relationship between quantitative imaging parameters and laboratory test results in all SLE patients. Partial correlation analysis was conducted after controlling for age, sex, BMI, medication use, and hypertension. The *p*-values reported for partial correlation analyses were uncorrected for multiple comparisons, as these analyses were considered exploratory.

To explore the potential classification performance of MRI quantitative parameters and clinical data in NPSLE, four candidate indicators were selected based on their clinical relevance and prior evidence from imaging and clinical studies: DTI-ALPS, NWI, WMH status, and SLEDAI. A multivariate binary logistic regression was performed to assess the contribution of each feature. Bootstrap resampling [1,000 samples, BCa 95% confidence interval (CI)] was used to assess the stability of the regression coefficients. Based on clinical and pathophysiological mechanisms, the model variables were grouped into three categories: (1) Clinical data: SLEDAI; (2) Conventional imaging features: WMH status; (3) MRI quantitative parameters: DTI-ALPS and NWI. Four multimodal classification models were constructed and compared: Model A: Combined (1) + (2) + (3); Model B: Combined (1) + (3); Model C: Combined (1) + (2); Model D: Combined (1) + DTI-ALPS only. Model discrimination was evaluated using the area under the receiver operating characteristic curve (AUC).

## 3 Results

### 3.1 Demographics, clinical, and imaging findings

A cohort of 60 patients with SLE (58 females) and 20 HC (18 females) were included in the analysis (*P* = 0.480). The SLE cohort was further divided into the Non-NPSLE group (*n* = 38) and the NPSLE group (*n* = 22). The median age of the HC, Non-NPSLE, and NPSLE groups were 39.5 (28∼51) years, 42.5 (32.5∼49.25) years, and 47 (32.5∼54.5) years (*P* = 0.554), with no significant differences in age or sex among the three groups. The median disease duration for the Non-NPSLE and NPSLE groups were 11 months and 17 months, with no significant difference between them (*P* = 0.053). We found that the NPSLE had significantly fewer positive cases for anti-Sm antibodies and anti-RNP antibodies compared to the Non-NPSLE (*P* = 0.021, *P* = 0.005). The NPSLE also showed significantly higher numbers of WMH (*P* = 0.037), TC (*P* = 0.015), HDL (*P* = 0.012) and SLEDAI (*P* = 0.019), compared to the Non-NPSLE. Among the 22 patients classified as NPSLE, neuropsychiatric manifestations included headache (*n* = 9), cognitive dysfunction (*n* = 4), mood disorder (*n* = 3), and seizure disorder (*n* = 1). In addition, five patients (*n* = 5) did not present with overt neuropsychiatric symptoms at the time of MRI acquisition. Neuroimaging abnormalities were observed in these cases, including multiple ischemic lesions (*n* = 2) and scattered WMH (+) (*n* = 3). Neuropsychiatric manifestations emerged shortly thereafter within the same disease course, including headache (*n* = 4, 1–4 days after imaging) and cognitive dysfunction (*n* = 1, 8 days after imaging). NPSLE attribution was determined through multidisciplinary clinical evaluation according to established neuropsychiatric lupus definitions, and imaging findings were not used as primary criteria for group allocation. Interobserver reliability was assessed using the ICC, with the results presented in Table 1. The clinical data of the patients are summarized in Table 2.

**TABLE 1:** Intraclass correlation coefficients.

Parameter	ICC	95%CI	ReaderA	ReaderB	*P*-value
DTI-ALPS	0.83	0.672,0.916	1.004 ± 0.058	1.005 ± 0.062	*P* < 0.001
LA	0.619	0.332,0.803	5.019 ± 1.234	5.175 ± 1.345	0.002
WA	0.847	0.712,0.922	0.750 ± 0.119	0.728 ± 0.108	*P* < 0.001
WT	0.868	0.745,0.935	7.558 ± 1.846	7.318 ± 1.776	*P* < 0.001
NWI	0.82	0.658,0.910	0.594 ± 0.060	0.592 ± 0.045	*P* < 0.001

Intraclass correlation coefficients [ICC (3,1)] with 95% confidence intervals for measurements by two independent observers (*n* = 60). ICC was calculated using a two-way mixed-effects model for absolute agreement. Reliability classification: excellent (ICC ≥ 0.75), good (ICC = 0.60–0.74), fair (ICC = 0.40–0.59), poor (ICC < 0.40).

**TABLE 2:** Clinical characteristics of enrolled participants.

Characteristic	Non-NPSLE	NPSLE	HC	*P*-value
Sex, (Female)	38 (37)	22 (21)	20 (18)	0.480
Age, (IQR)	42.5 (32.5∼49.25)	47 (32.5∼54.5)	39.5 (28∼51)	0.554
BMI, (IQR)	23.41 (21.25∼25.04)	23.44 (20.69∼25.26)	23.32 (20.9∼25.18)	0.725
Duration (months), (IQR)	11 (7.0∼17.25)	16.5 (12.5∼20)		0.053
ANA (+), n (%)	21 (55.3%)	12 (54.5%)		0.957
Anti-dsDNA (IU/mL), (IQR)	5.58 (5.58∼35.80)	12.13 (3.89∼16.73)		0.724
Anti-dsDNA (+), n (%)	12 (31.6%)	5 (22.7%)		0.463
Anti-Sm (IU/mL), (IQR)	2.42 (2.42∼16.85)	3.35 (1.02∼7.59)		0.969
Anti-Sm (+), n (%)	7 (18.4%)	1 (4.5%)		0.021
ANua (IU/mL), (IQR)	1.71 (1.26∼6.05)	3.45 (1.97∼5.83)		0.116
ANua (+), n (%)	1 (2.6%)	1 (4.5%)		0.274
Anti-RNP (IU/mL), (IQR)	1.71 (0.82∼36.42)	5.37 (1.18∼9.55)		0.951
Anti-RNP (+), n (%)	12 (31.6%)	0 (0%)		0.005
C3 (g/L), (IQR)	0.97 (0.84∼1.10)	0.94 (0.85∼1.03)		0.575
Reduced C3, n (%)	5 (13.2%)	2 (9.1%)		0.191
C4 (g/L), (IQR)	0.18 (0.13∼0.23)	0.19 (0.14∼0.23)		0.645
Reduced C4, n (%)	8 (21.1%)	5 (22.7%)		0.618
IgA (g/L), (IQR)	2.18 (1.64∼2.66)	2.47 (1.44∼3.23)		0.286
IgE (IU/mL), (IQR)	31.48 (20.84∼53.46)	26.45 (18.64∼71.25)		0.565
IgM (g/L), (IQR)	0.77 (0.52∼1.01)	0.73 (0.51∼1.01)		0.495
IgG (g/L), (IQR)	12.21 (10.17∼15.43)	12.03 (9.24∼12.78)		0.338
TC (mmol/L), (IQR)	4.19 (3.79∼4.62)	4.7 (4.07∼5.58)		0.015
TG (mmol/L), (IQR)	1.47 (1.09∼1.70)	1.8 (1.28∼2.25)		0.082
HDL (mmol/L), (IQR)	1.26 (1.02∼1.44)	1.49 (1.34∼1.7)		0.012
LDL (mmol/L), (IQR)	2.44 (2.02∼2.78)	2.76 (2.13∼3.26)		0.106
SLEDAI, (IQR)	8 (2∼12.5)	13 (7.5∼22.5)		0.019
WMH (+), n (%)	11 (28.9%)	14 (63.6%)		0.037
Hypertension, n (%)	5 (13.2%)	2 (9.1%)		1.000
Corticosteroid use, n (%)				0.246
>10 mg/day	32 (84.2%)	21 (95.5%)		
≤ 10 mg/day	5 (15.8%)	1 (4.5%)		
Immunosuppressive agents, n (%)				0.164
Yes	11 (28.9%)	11 (50%)		
No	27 (71.1%)	11 (50%)		

BMI, Body Mass Index (Weight/Height^2^); ANA, Anti-nuclear Antibody; ANuA, anti-nucleosome antibodies; Anti-RNP, Antiribonucleoprotein Antibody; TC, Total Cholesterol; TG, Triglycerides; HDL, High-Density Lipoprotein; LDL, Low-Density Lipoprotein; SLEDAI, Systemic Lupus Erythematosus Disease Activity Index; WMH, White Matter Hyperintensities. Corticosteroid dose was classified as high or low using a threshold equivalent to 10 mg/day of prednisone. *P*-value (statistical significance was set at *P* < 0.05).

### 3.2 Quantitative parameters group differences

Significant differences were observed among the three groups for DTI-ALPS values (*H* = 28.196, *P* < 0.001, η^2^ = 0.340), LA (*H* = 10.672, *P* = 0.005, η^2^ = 0.113), and NWI (*H* = 14.386, *P* = 0.001, η^2^ = 0.161), as shown in Table 3. Pairwise comparisons revealed that the HC group had a significantly lower NWI compared to the Non-NPSLE group (*P* = 0.029, MD = –0.04, 95% CI = –0.07 ∼ –0.01). The HC also had significantly higher DTI-ALPS values (*P* < 0.001, MD = 0.09, 95% CI = 0.05 ∼ 0.12) and LA (*P* = 0.023, MD = 1.05, 95% CI = 0.09 ∼ 1.66) compared to the NPSLE, while NWI was significantly lower in the HC group (*P* = 0.001, MD = –0.06, 95% CI = –0.10 ∼ –0.02). Furthermore, the Non-NPSLE group had significantly higher DTI-ALPS values (*P* < 0.001, MD = 0.08, 95% CI = 0.05 ∼ 0.11) and LA (*P* = 0.040, MD = 0.71, 95% CI = 0.02 ∼ 1.38) compared to the NPSLE group. Detailed results are presented in Table 4.

**TABLE 3:** Overall group differences.

Parameter	HC (*n* = 20)	Non-NPSLE (*n* = 38)	NPSLE (n = 22)	*H*-value	*P*-value	ε ^2^
DTI-ALPS	1.02 (1.01∼1.04)	1.01 (1.00∼1.05)	0.93 (0.93∼0.95)	28.196	*P* < 0.001	0.340
LA	5.73 (4.97∼5.98)	5.39 (4.63∼6.13)	4.68 (4.20∼5.02)	10.672	0.005	0.113
WT	0.70 (0.63∼0.74)	0.71 (0.66∼0.78)	0.73 (0.65∼0.79)	1.010	0.603	0
WA	6.88 (6.02∼7.60)	7.39 (6.46∼7.90)	7.50 (6.35∼7.94)	1.285	0.526	0
NWI	0.55 (0.53∼0.58)	0.59 (0.56∼0.62)	0.61 (0.57∼0.63)	14.386	0.001	0.161

H, Kruskal-Wallis H test statistic; ε^2^, Epsilon squared; a measure of effect size indicating the magnitude of differences between groups. The effect size was interpreted against the following benchmarks: small (ε^2^ ≥ 0.01), medium (ε^2^ ≥ 0.06), and large (ε^2^ ≥ 0.14). DTI-ALPS Diffusion Tensor Image—Analysis along the Perivascular Space. LA, Lumen Area; WA, Wall Area; WT, Wall Thickness; NWI, Normalized Wall Index.

**TABLE 4:** Quantitative parameters group differences.

Parameter	HC vs. Non-NPSLE	HC vs. NPSLE	Non-NPSLE vs. NPSLE
*P*-value, MD, 95%CI			
DTI-ALPS	0.472, 0.01, –0.26∼0.36	*P* < 0.001, 0.09, 0.05∼0.12	*P* < 0.001, 0.08, 0.05∼0.11
LA	0.322, 0.34, –0.52∼0.87	0.023, 1.05, 0.09∼1.66	0.040, 0.71, 0.02∼1.38
NWI	0.029, –0.04, –0.07∼-0.01	0.001, –0.06, –0.10∼-0.02	0.260, –0.02, –0.05∼-0.01

MD, median difference; CI, confidence interval; *P*-value (statistical significance was set at *P* < 0.05).

### 3.3 Laboratory and quantitative parameters correlation analyses

Correlation analysis showed that DTI-ALPS values were significantly negatively correlated with TC (*r* = *-*0.389, *P* = 0.002). WT was significantly positively correlated with IgG (*r* = 0.270, *P* = 0.037), and significantly negatively correlated with IgE (*r* = –0.292, *P* = 0.023) and LDL (*r* = –0.308, *P* = 0.017). WA was also significantly negatively correlated with disease duration (*r* = –0.270, *P* = 0.037), IgE (*r* = –0.309, *P* = 0.016), and LDL (*r* = –0.280, *P* = 0.030). Additionally, LA was significantly negatively correlated with disease duration (*r* = –0.391, *P* = 0.002), LDL (*r* = –0.308, *P* = 0.017), and TC (*r* = –0.262, *P* = 0.044). NWI was significantly positively correlated with anti-dsDNA antibodies (*r* = 0.273, *P* = 0.035), and significantly positively correlated with HDL (*r* = 0.310, *P* = 0.016). Partial correlation analysis revealed that DTI-ALPS values were significantly negatively correlated with TC and IgA (*r* = –0.308, *P* = 0.025; *r* = –0.301, *P* = 0.028), WT, WA and LA were all significantly negatively correlated with LDL (*r* = –0.273, *P* = 0.045; *r* = –0.271, *P* = 0.048; *r* = –0.293, *P* = 0.032), LA was significantly negatively correlated with disease duration (*r* = –0.474, *P* < 0.001), while NWI was significantly positively correlated with HDL (*r* = 0.284, *P* = 0.025). As shown in Table 5.

**TABLE 5:** Partial correlation analysis.

Parameter	ZTC	ZLDL	ZHDL	ZIgA	ZDuration
Spearman r, *P*-value					
ZDTI-ALPS	0.023	0.09	0.616	0.026	0.775
	–0.310	–0.235	–0.070	–0.303	–0.040
ZWT	0.194	0.045	0.164	0.639	0.321
	0.-180	–0.273	0.192	–0.065	–0.138
ZWA	0.191	0.048	0.396	0.310	0.131
	–0.181	–0.271	0.118	–0.141	–0.208
ZLA	0.085	0.032	0.170	0.174	*P* < 0.001
	–0.236	–0.293	–0.189	–0.188	–0.394
ZNWI	0.515	0.340	0.038	0.589	0.376
	–0.091	–0.132	0.284	0.075	0.123

Partial correlation analysis assessing the relationships between key serological markers, clinical parameters, and neurovascular imaging metrics, after controlling for age, sex, body mass index, medicine, and history of hypertension. The partial correlation coefficient (r) ranges from -1 to +1, indicating perfect negative to perfect positive correlation, respectively. *P*-value (statistical significance was set at *P* < 0.05). All analyzed metrics (including TC, DTI-ALPS, IgA, IgE, WT, LDL, WA, LA, Duration, and NWI) were standardized as Z-scores to statistical analysis.

### 3.4 Multimodal classification model

**FIGURE 2:**
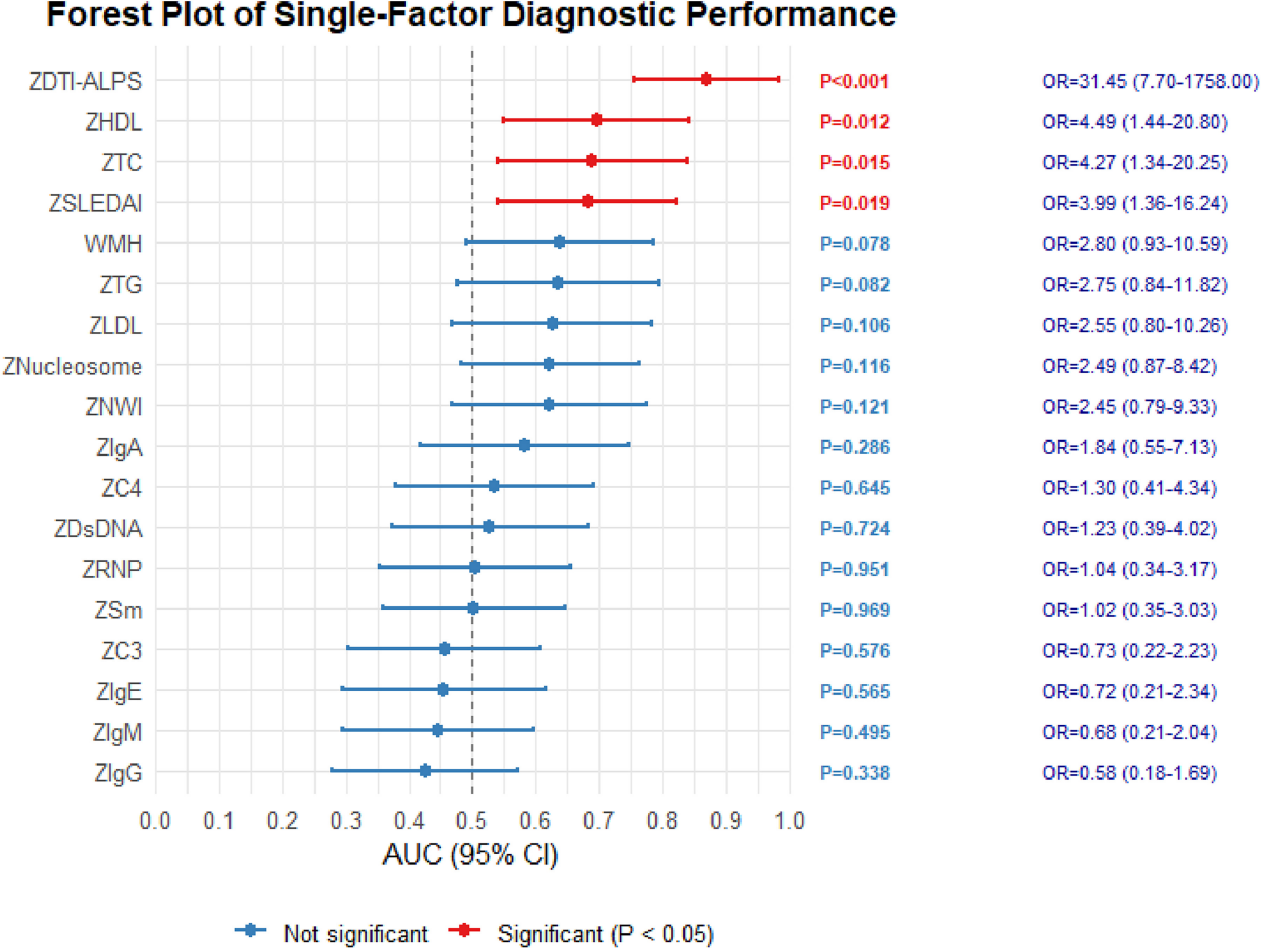
Forest plot of single-factor classification performance. The image presents the results of a univariate analysis examining the associations between multiple clinical indicators, quantitative imaging parameters, and NPSLE status. This figure illustrates the classification performance and statistical significance of each variable using odds ratios, confidence intervals, and *P*-values.

**FIGURE 3:**
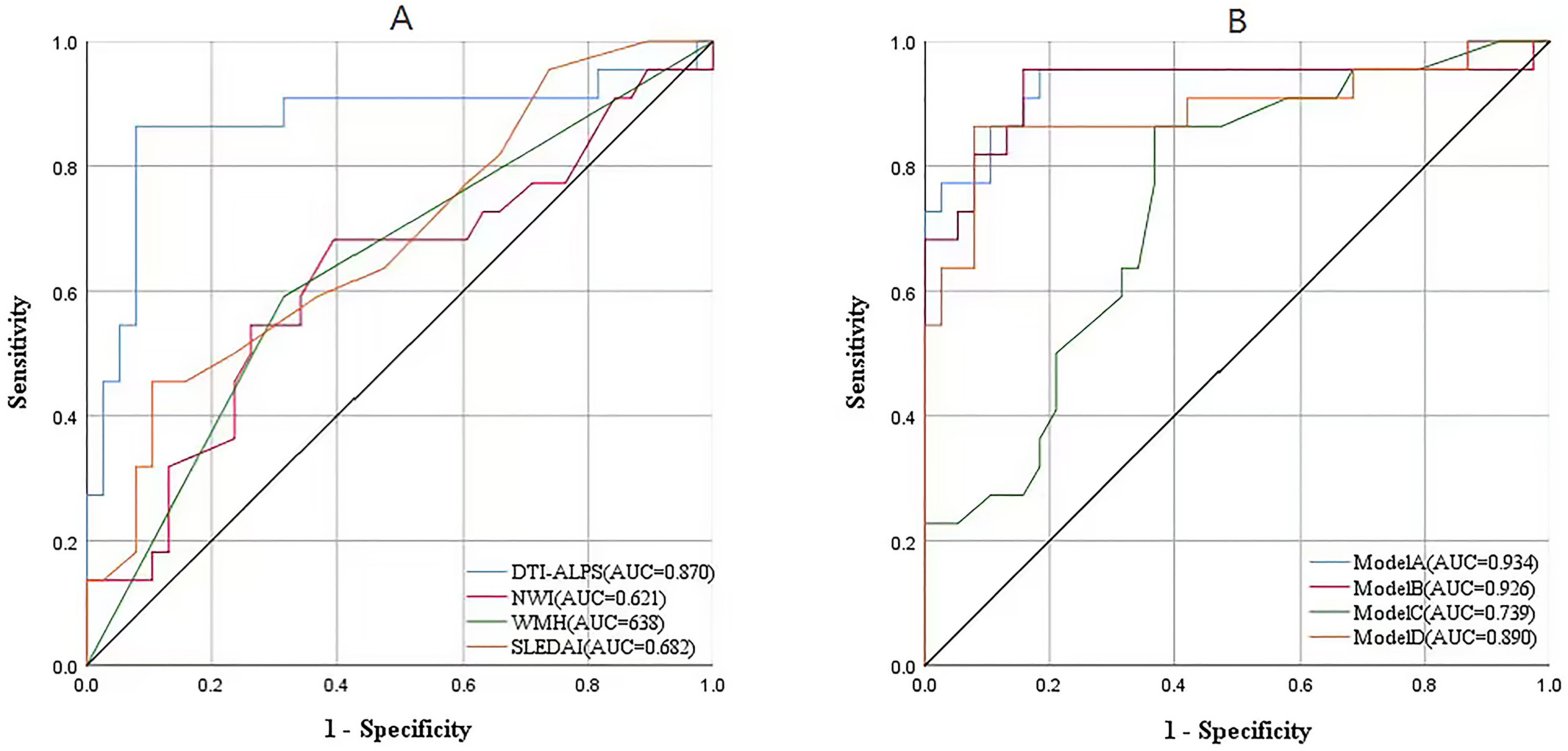
Comparison of the performance of different exploratory models. **(A)** The classification AUC values of individual features. **(B)** The exploratory classification performance of the four models: Model A incorporates SLEDAI, WMH, DTI-ALPS, and NWI; Model B incorporates SLEDAI, WMH, and DTI-ALPS; Model C incorporates SLEDAI and WMH. Model D incorporates SLEDAI and DTI-ALPS.

The multivariate binary logistic regression analysis of feature contributions is shown in Figure 2. DTI-ALPS (*P* < 0.001, 95% CI = 7.70∼1758, OR (odds ratio) = 31.45), NWI (*P* = 0.121, 95% CI = 0.55∼7.13, OR = 2.45), WMH status (*P* = 0.078, 95% CI = 0.84∼11.82, OR = 2.80), and SLEDAI (*P* = 0.019, 95% CI = 1.36∼16.24, OR = 3.99). DTI-ALPS and SLEDAI were significantly associated with NPSLE in the multivariate model, whereas NWI and WMH status were included as candidate predictors based on their clinical and imaging relevance. The classification performance of the four multimodal classification models was evaluated as follows: Model A (1)+(2)+(3) had an AUC of 0.933 (*P* < 0.001, 95% CI = 0.853∼1.000), Model B (1)+(3) had an AUC of 0.925 (*P* < 0.001, 95% CI = 0.848∼1.000), Model C (1)+(2) had an AUC of 0.731 (*P* = 0.002, 95% CI = 0.602∼0.860), and Model D (1)+DTI-ALPS had an AUC of 0.890 (*P* < 0.001, 95% CI = 0.788∼0.992). Significant differences in AUC were observed between Model A and Model C (*P* < 0.001), as well as between Model B and Model C (*P* = 0.001). To further assess model robustness, bootstrap resampling demonstrated that the direction of effects for key predictors, particularly DTI-ALPS, remained consistent with the original model, supporting the stability of the regression coefficients despite the limited sample size. Results are shown in Figure 3.

## 4 Discussion

This study is the first to combine multimodal MRI approaches, including DTI and HR-VWI, to explore cerebrovascular pathological changes and brain glymphatic function in SLE patients. By constructing a multimodal classification model, we evaluated the role of MRI quantitative parameters in characterizing neurovascular imaging patterns and neuropsychiatric involvement in NPSLE.

NWI provides an objective evaluation of the vascular wall across different ages and anatomical locations (Xiong et al., 2025). The M1 segment, reported to be frequently involved in NPSLE and consistent with the findings of Zhao et al. (Zhao et al., 2025), was selected as a representative region for evaluating cerebrovascular pathology.

The brain glymphatic system, responsible for maintaining the brain’s internal homeostasis, could be disrupted when cerebral vascular walls thicken and vascular lumens decrease. This may lead to poor interstitial fluid (ISF) and Cerebrospinal Fluid (CSF) exchange, thereby impairing material transfer (Moses et al., 2023), as shown by decreased DTI-ALPS values.

Our study revealed that the TC and SLEDAI scores in the NPSLE group are higher than in the Non-NPSLE group, from HC to Non-NPSLE, and then to NPSLE, both DTI-ALPS and LA gradually decreased significantly, NWI in Non-NPSLE is significantly higher than in HC, LA was significantly negatively correlated with LDL and disease duration, TC and IgA were significantly negatively correlated with DTI-ALPS values. These findings suggest a potential link in SLE patients from immune and lipid metabolic disturbances to vascular changes and glymphatic dysfunction in the brain. Hyperactive immune responses and abnormal lipid metabolism in SLE patients may contribute to this process with elevated IgA and high TC exacerbating vascular inflammation and angiosclerosis (Salomonsson et al., 2023). These immunometabolic abnormalities lead to vascular remodeling, reducing LA and increasing NWI, which hinders glymphatic exchange, reflected in lower DTI-ALPS values. This result is consistent with the findings of Mo et al. (2025). Such glymphatic impairment may be associated with neurotoxic accumulation and could contribute to neuropsychiatric manifestations and higher SLEDAI scores. In our NPSLE cohort, headache was the predominant clinical manifestation. As the cerebrovascular lesions in SLE patients progress, cognitive dysfunction and symptoms of intracranial hemorrhage may subsequently develop (Aringer et al., 2019). Patients with a longer disease duration demonstrated more pronounced cumulative damage to the vascular wall and glymphatic function, consistent with the finding of Nomura et al. (1999). Some patients classified as NPSLE did not show overt neuropsychiatric symptoms at the time of imaging, with asymptomatic status defined based on clinical data collected at MRI acquisition, highlighting the clinical heterogeneity of NPSLE and suggesting that MRI abnormalities may occur independently of current clinical manifestations. Early identification of NPSLE-related neurovascular and glymphatic abnormalities may have clinical implications, as it could support earlier immunomodulatory intervention and closer neurological monitoring, thereby helping to prevent cumulative or irreversible central nervous system damage.

Our study revealed that the HDL level in the NPSLE group was higher than in the Non-NPSLE group and HDL was significantly positively correlated with NWI. HDL has been reported to exert anti-inflammatory and vasoprotective effects (Kim, 2021). This observation may reflect a compensatory vascular response but should be interpreted cautiously given the observational study design. Partial correlation analyses showed that both WT and WA were significantly negatively correlated with LDL. The positivity rates of anti-Sm and anti-RNP antibodies were higher in the non-NPSLE than in the NPSLE patients. As medication exposure did not differ significantly between groups and the sample size was limited, these antibody findings are presented as descriptive associations rather than evidence of medication-related or pathophysiological effects, and may reflect cohort heterogeneity requiring validation in larger studies.

When constructing the multimodal classification model for NPSLE, we did not rely solely on statistical significance for feature selection. Instead, DTI-ALPS and NWI were included as key parameters consistent with our research objectives. To compare the contribution of DTI and HR-VWI derived quantitative parameters with conventional imaging, the WMH status was also incorporated. Model A (AUC = 0.933) showed encouraging classification performance in this exploratory cohort, significantly outperforming Model C (AUC = 0.731), thereby suggesting the potential contribution of DTI-ALPS and NWI in distinguishing neurovascular imaging patterns in NPSLE within this exploratory cohort. The minimal difference between Model A and Model B (AUC = 0.933 vs. 0.925) suggests that WMH features contribute relatively little to classification performance in this cohort. The relatively lower contribution of WMH to classification performance in the present study may partly relate to the binary categorization strategy, which does not capture lesion burden or severity. More granular grading approaches, such as the Fazekas scale, may provide additional sensitivity and could potentially improve the characterization of conventional MRI features in future studies. This finding aligns with clinical observations that some NPSLE patients do not exhibit WMH (+) on conventional MRI (Liu et al., 2018; Zhao et al., 2018), underscoring its limited role in assessing the condition.

Analysis of NWI and DTI-ALPS values revealed cerebrovascular alterations and glymphatic dysfunction in SLE patients. Comparing Model B and Model D (AUC = 0.925 vs. 0.890) indicates that DTI-ALPS plays a more prominent role than NWI in differentiating NPSLE within this classification framework, though NWI remains a valuable auxiliary parameter. In addition to the impact of vascular structural changes on exchange efficiency and waste clearance, elevated TC increases blood viscosity and impairs metabolic waste removal, consequently, DTI-ALPS values more sensitively reflect lesion severity than NWI, making it a superior biomarker for early detection. In brief, combining conventional imaging with MRI quantitative parameters may improve exploratory classification performance and contributes to a deeper understanding of NPSLE pathophysiology from an imaging perspective. These findings support the relative stability of the regression coefficients despite the limited sample size.

Zhao et al. have developed an intracranial vessel wall enhancement scoring system for SLE patients utilizing enhanced HR-VWI technology (Zhao et al., 2025), although analogous imaging techniques were employed, these studies relied on contrast agents. Bai et al. employed DTI combined with 3D-T1 or magnetization transfer imaging (MTI) and revealed associations between alterations in brain structural networks and neuropsychiatric symptoms in SLE (Bai et al., 2025; Ercan et al., 2015). Despite differences in methodology, consistent findings have demonstrated significant imaging differences between non-NPSLE and NPSLE groups. As a biomarker for evaluating the glymphatic function, DTI-ALPS has been used in Alzheimer’s disease and mild cognitive impairment (Harrison et al., 2020; Xie et al., 2013). Its application in NPSLE in this study further demonstrates its potential.

In summary, this study identified significant differences in cerebrovascular microstructure and glymphatic function among NPSLE patients, Non-NPSLE patients and HC, providing preliminary imaging insights into cerebrovascular alterations and glymphatic dysfunction associated with NPSLE. Given the lack of specificity in clinical and conventional imaging manifestations of NPSLE (Van Der Kolk et al., 2015), Our multimodal classification modeling suggests potential roles of MRI quantitative parameters in understanding disease mechanisms.

Several limitations should be acknowledged. First, as a single-center prospective study, the relatively limited sample size may affect the generalizability of the findings. Although bootstrap resampling suggested consistent effect directions for key predictors, the exploratory nature of the modeling approach should be considered when interpreting the results. Second, during patient recruitment, hypertension was not strictly excluded. Although the statistical analysis accounted for the impact of hypertension on the results, residual confounding effects cannot be completely excluded. Third, due to the spatial resolution constraints of vessel wall imaging and non-invasive MRI techniques, only major intracranial arteries were evaluated, precluding assessment of smaller vessel changes. Future studies involving larger, multicenter cohorts are warranted to validate these findings. Additionally, combining MRI techniques that assess BBB permeability without contrast agents may provide further insights into the pathophysiology of SLE.

## Data Availability

The raw data supporting the conclusions of this article will be made available by the author, without undue reservation.
